# Extracellular Vesicles from Plasma of Patients with Glioblastoma Promote Invasion of Glioblastoma Cells Even After Tumor Resection

**DOI:** 10.3390/biomedicines12122834

**Published:** 2024-12-13

**Authors:** Ekaterina N. Lyukmanova, Artem V. Kirichenko, Igor A. Medyanik, Konstantin S. Yashin, Mikhail P. Kirpichnikov, Maxim L. Bychkov

**Affiliations:** 1 Faculty of Biology, Shenzhen MSU-BIT University, Shenzhen 518172, China; 2 Shemyakin-Ovchinnikov Institute of Bioorganic Chemistry, Russian Academy of Sciences, 119997 Moscow, Russia; bittert@mail.ru (A.V.K.); kirpichnikov@inbox.ru (M.P.K.); 3 Moscow Center for Advanced Studies, 123592 Moscow, Russia; 4 Interdisciplinary Scientific and Educational School of Moscow University «Molecular Technologies of the Living Systems and Synthetic Biology», Faculty of Biology, Lomonosov Moscow State University, 119234 Moscow, Russia; 5 Department of Neurosurgery, Privolzhsky Research Medical University, 603005 Nizhny Novgorod, Russia; med_neuro@inbox.ru (I.A.M.); jashinmed@gmail.com (K.S.Y.)

**Keywords:** glioblastoma, extracellular vesicles, astrocytes, invasion, AKT, JNK, p38, E/N cadherin switch, cytokines, adhesion molecules, inflammatory molecules

## Abstract

**Background**: Glioblastoma (GB) is a highly aggressive tumor, whose progression is mediated by secretion of extracellular vesicles (EVs), which can pass the brain–blood barrier and be found in the plasma. Here, we performed a comparative analysis of the effects of EVs from the plasma of healthy donors (hEVs) and GB patients before (bEVs) and after (aEVs) tumor surgical resection on invasion of normal astrocytes and GB cells. **Methods**: We performed the transwell invasion assay, analyzed MAP kinases activation by Western blotting, studied SNAI1/SNAI2 cellular localization by confocal microscopy, measured cadherins expression by flow cytometry, and analyzed secretion of cytokines, which regulate migration and inflammation, by immunoassay. **Results**: hEVs did not affect invasion of astrocytes and GB cells, there was down-regulated cadherins expression in astrocytes, while there was increased E- and N-cadherin expression in GB cells. hEVs increased the secretion of inflammation and adhesion regulators both in astrocytes and GB cells. bEVs enhanced the invasion of GB cells but not of astrocytes via MAP AKT, JNK1/2/3, and p38 kinases activation, stimulated the clasterization of SNAI1 in the GB cell nucleus, promoted an E/N cadherin switch, and caused the secretion of inflammation and adhesion regulators in astrocytes and GB cells. aEVs exhibited the most of pro-oncogenic effects of bEVs (stimulation of GB cell invasion, SNAI1 nuclear localization, JNK1/2/3 activation, E/N cadherin switch, and secretion of inflammation and adhesion regulators in astrocytes and GB cells). However, aEVs effects were less pronounced than those of bEVs. **Conclusions**: In our study, we revealed common and different effects of plasma-derived hEVs, aEVs, and bEVs. hEVs can stimulate some pro-oncogenic effects in GB cells. Being less tumorigenic then bEVs, aEVs are still able to promote invasion of GB cells, probably remaining after tumor resection.

## 1. Introduction

Glioblastoma (GB) is the most aggressive brain tumor characterized by rapid growth, metastasis, establishment of an inflammatory microenvironment and high heterogeneity [1]. To support tumor progression, GB cells secrete into the environment numerous pro-oncogenic cytokines and adhesion molecules [2], as well as extracellular vesicles (EVs) containing pro-oncogenic factors [3]. The transport of these vesicles through the body allows tumor cells to ‘communicate’ with each other and to promote tumor growth, metastasis, and chemoresistance [4]. GB-derived EVs (GDEVs) influence the cells surrounding a tumor [5], promote tumor vascularization [6], and transfer pro-oncogenic factors into the tumor endothelium [6].

EVs from cultured GB cells promote various effects in the brain cells: disruption of neuronal synchronization [7], regulation of proliferation and activation of immune cells in the brain [5,8,9], induction of malignant transformation of normal astrocytes [10], enhancement of proliferation, migration and invasion of GB cells [11,12], and stimulation of angiogenesis [12]. GDEVs also regulate secretion of various cytokines and adhesion molecules by immune cells [13,14] and normal astrocytes [15]. EVs are the central players in regulation of the inflammatory microenvironment of GB [13]. GDEVs can cross through the blood–brain barrier (BBB) in both directions [6], so they can be detected in the bloodstream [16,17] and cerebrospinal fluid (CSF) [18,19,20] of patients. In addition, GDEVs can disrupt the BBB function by activation of endothelial permeability regulator semaphorin-3A [21]. EV concentration in the plasma of GB patients is higher than in the plasma of healthy donors, and decreases upon tumor resection and radiation/temozolomide therapy [22,23]. GDEVs from the plasma can transport different pro-oncogenic proteins and RNAs, for example, mRNA encoding alkylpurine-DNA-N-glycosylase (*APNG*) and O^6^-methylguanine DNA methyltransferase (*MGMT*), which mediate a resistance to the temozolomide therapy in GB patients and GB cells [24]. GDEVs from the plasma and CSF also contain a high level of pro-oncogenic miRNA-21, which mediates GB growth and invasion [25]. Moreover, in comparison with EVs derived from the plasma of healthy donors, plasma GDEVs contain a higher level of hypoxia-regulated proteins (such as VEGF and metalloprotease inhibitor TIMP), which regulate GB vascularization [26], pro-oncogenic integrin β1, hyaluronic acid receptor CD44, cytoskeleton protein CD146, complement protein C1Qa, and histone H3 [27]. Another aspect of the effects of plasma GDEVs is immunosuppression by the down-regulation of proliferation of tumor-inhibitory T cells [13]. Plasma GDEVs contain immunosuppressive miRNAs (miR-1246, miR-29a, miR-10a, etc.) [28] and high levels of PD-L1, which drives differentiation of monocytes towards the non-classical immunosuppressive phenotype [29]. On the other hand, EVs secreted by tumor-surrounding microglia cells can restrict GB cells’ metabolism via miR-124 and reduce GB growth in vivo [30]. Factors delivered by GDEVs from CSF (mutated variant of EGFR, -EGFR vIII, and pro-oncogenic miR-21, miR-24, miR-103, and miR-125) can stimulate GB proliferation, invasion, and angiogenesis [31]. Similar to plasma GDEVs, GDEVs from CSF suppress the presentation of GB antigens by DC cells via transfer of adhesion protein galectin 9 [32].

GDEVs from both plasma and CSF can be used for tumor diagnosis or serve as biomarkers of its progression [8,33,34,35]. Several clinical trials have been conducted to explore the potential of GDEVs as markers of GB progression and response to the therapy. For example, the mRNA level of IL8 and TGFβ in plasma GDEVs correlates with the immunological response of GB patients to dendritic cell-based vaccines [36]. Some molecules from GDEVs of CSF (cytoskeletal protein MYO1C, IDH1-mutant mRNA, miR-9, miR-30-3p, and miR-1298-5p) can serve as markers for GB diagnostics and monitoring [37]. Another recent clinical trial showed the perceptiveness of the serum GDEVs analysis for the classification and monitoring of CNS tumors [38]. Furthermore, due to their low immunogenicity, EVs secreted by mesenchymal stem cells [39] or EVs loaded with therapeutic agents [40] can be used for targeted delivery of therapeutic substances to GB. However, presently there is no information about ongoing trials of EVs as therapeutic agents on ClinicalTrials.gov. Additionally, EVs can be loaded with fluorescent agents and serve as tracking tools [41].

Thus, GDEVs are the central transmitters of pro-oncogenic signals both into the tumor itself, surrounding tissues, and through the body. While many aspects of the action of EVs from cultured cells in GB are known, the effects of EVs from the plasma of healthy and GB patients on glial cells have not been compared. Moreover, although secretion of various inflammatory adhesion factors by GB cells is recognized as an important mechanism of tumor progression [2], the effect of EVs on the secretion of these molecules by GB cells also remains unclear.

Here, we investigated the influence of EVs isolated from the plasma of healthy donors and patients with GB (before and after tumor resection) on invasion of the normal astrocytes and GB cells, and revealed the mechanisms mediating these effects.

## 2. Materials and Methods

### 2.1. Patients

The plasma from healthy donors (n = 8) and patients with GB (n = 8, clinically assessed as histological grade IV, *IDH* wild-type) was obtained from patients of the Clinic of Privolzhsky Research Medical University (Nizhny Novgorod, Russia) after informed consent and approval of the ethics committee (protocol № 12 from 25 June 2019). The plasma before and after resection was collected from the same patient on the previous and next days after tumor resection, respectively.

### 2.2. Isolation and Characterization of EVs

EVs were isolated from the plasma of patients using the Total Exosome Isolation kit (4484450, Thermo Fisher Scientific, Waltham, CA, USA) according to the manufacturer instructions. An amount of 0.5 mL of the plasma was centrifuged 2 times (2000× *g* and 10,000× *g* for 20 min, RT), diluted 2 times with PBS, and the exosome precipitation reagent was added. After that, plasma was incubated at 37 °C for 1 h. Then, precipitated EVs were washed from the resting plasma by centrifugation (10,000× *g*, RT). The supernatant was aspirated and discarded, while the EV-containing pellet was resuspended in PBS and centrifugated (10,000× *g*, RT). The EV-containing supernatant was collected, filtered through 0.2 µm PVDF syringe filter (Merck, Darmstadt, Germany), EVs were aliquoted, frozen at −150°C, and used for characterization and assays. EVs were not subjected to freeze–thaw cycles.

**Figure 1:**
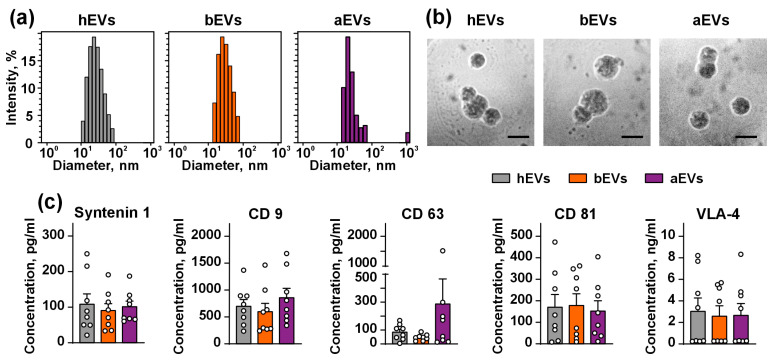
Characterization of EVs from the plasma of healthy donors and GB patients before and after tumor resection: (**a**) representative distribution of EVs according to the size determined by dynamic light scattering; (**b**) representative pictures of EVs according to scanning EM (scale = 50 nm); (**c**) expression of EV markers, no cytochrome C was detected in all studied EVs. Data presented as protein concentration ± SEM (n = 8).

EVs were characterized using dynamic light scattering (DynaPro Titan, Wyatt Technology, Golata, CA, USA) with 828 nm laser and scattering angle 90°. Particles with diameters in the range of 20–120 nm were detected in EV suspension (Figure 1a). The size of EVs was in agreement with the previously published data [42]. It should be noted that plasma-derived EVs are smaller than those from cultured cells [43] and have the higher size distribution [44]. EVs were visualized using scanning electron microscope REM 200 (Moscow, Russia) under 15 kW power. Mainly, EVs with an average diameter ~50 nm were observed (Figure 1b). Expression of vesicular molecular markers in EVs were accessed using the ProcartaPlex™ Human Exosome Characterization Panel (EPX060-15845-901, Thermo Fisher Scientific). All samples contained CD9, CD63, CD81, syntenin-1, and VLA4, but not cytochrome C (Figure 1c). No statistical differences in the protein content were observed between the samples. All experiments were performed at the EV concentration of 50 μg/mL, which was in a range of that found in the plasma of patients with GB (20–100 µg/mL) [45].

To confirm a relevance of the Total Exosome Isolation kit for EV isolation, we compared the properties of EVs derived from the plasma of three patients and isolated them by the method described above and by widely used ultracentrifugation [46]. In both batches, we did not find a statistically significant difference in EVs’ marker expression including CD9 (Figure S1). Nevertheless, similar to [47], some difference in the size distribution of EVs isolated by different techniques was observed (Figure S1). We speculate that this difference may be due to high plasma EV heterogeneity. The choice of the Total Exosome Isolation Kit is also supported by the previous reports on EV isolation by its use for different in vitro [48,49,50,51] and in vivo [52,53] assays. Notably, the main study was performed using EVs isolated from the plasma of other patients.

### 2.3. Cell Cultivation and Isolation of Primary Astrocytes

Authenticated human GB U251 MG cells obtained from the Russian vertebrate cell culture collection (Institute of Cytology RAS, St. Petersburg, Russia) were grown in the IMDM medium (PanEco, Moscow, Russia) supplemented with 10% of FCS (Corning Corp., Corning, CA, USA). GB cell line GBM 011 was kindly provided by M.S. Pavlyukov (IBCH RAS, Moscow, Russia). GBM 011 cells were obtained from the patient of N.N. Burdenko National Medical Research Center of Neurosurgery (Moscow, Russia) after informed consent and were characterized previously [54]. GBM 011 cells were cultured for no longer than 10 passages in the DMEM/F12 medium supplemented with MACS NeuroBrew-21 supplement (Miltenyi Biotec, Gladbach, Germany, 10 mL of supplement per 500 mL of cell medium), 2.5 μg/mL heparin, 20 ng/mL bFGF, and 20 ng/mL EGF (all compounds were from Sigma-Aldrich, St. Louis, MO, USA). EGF and bFGF were added twice a week and the culture medium was changed every 7 days.

Rat cortical astrocytes were isolated as in [55] and grown in the DMEM/F12 (PanEco)/10% FCS medium with addition of MACS NeuroBrew-21 supplement. Forty-two-day-old astrocytes were used for the experiments. GFAP expression in the astrocytes was confirmed by flow cytometry.

All cells were monthly tested for mycoplasma contamination (Mycoreport kit, Evrogen, Moscow, Russia).

### 2.4. Transwell Invasion Assay

The transwell assay, in which cells migrate through 8 µm pores of polystyrene membrane, was used to study cell invasion. Cells were seeded in migration chambers in 24-well plates (SPL Lifesciences, Pocheon, Republic of Korea; 2 × 10^5^ cells/well), immediately treated with 50 µg/mL of EVs and incubated for 72 h without media change (the incubation time was chosen so that the cells did not die at the end of the experiment). Then, the cells migrating through the pores were photographed (100× magnification, CloneSelect Imager, Molecular Devices, San Jose, CA, USA). The number of invaded cells was quantified using the find maxima option of the ImageJ 1.54f software (NIH, Bethesda, MD, USA). For comparison of action of EVs from U251 MG cells (isolated as in [46]) and plasma EVs on U251 MG cells, the Cell Migration/Chemotaxis assay (ab235673Abcam, Cambridge, UK) was performed according to the manufacturer instructions.

### 2.5. Western Blotting

Cells (3 × 10^5^ cells per well of a 6-well plate) were treated with EVs (50 µg/mL) for 72 h, lysed in the RIPA buffer containing SIGMAFAST protease inhibitor cocktail (Sigma-Aldrich), diluted in the PAGE loading buffer and subjected to gel electrophoresis. The proteins were then transferred to nitrocellulose membranes (Bio-Rad, Hercules, CA, USA) which then were blocked for 1 h with 5% BSA in TBST. After that, the membranes were incubated with the rabbit primary antibodies to pAKT (S473) (1:4000, GB150002, ServiceBio, Wuhan, China), pERK1/2 (T202/204; T185/Y187) (1:1000, GB113492, ServiceBio), pJNK1/2/3 (T183/Y185) (1:1000, GB12018, ServiceBio), and pp38 (T180/Y182) (1:1000, GB1133880, ServiceBio) overnight at 4 °C. Note that pJNK and pp38 were assayed on the same membranes: pJNK was the first, and pp38 followed after membrane stripping. For antibodies’ wash-out, the membranes were incubated in the buffer (50 mM Tris, pH 6.8, 1% SDS, 0.8% β-mercaptoethanol) at 55 °C for 45 min, washed under running tap water for 5 min, incubated with TBST for 5 min, and processed from the blocking stage. Expression of pAKT and pERK was analyzed on one membrane, which was cut near 50 kDa. After incubation with the primary antibodies, the membranes were washed 3 times in TBST and incubated with anti-rabbit HRP-conjugated antibodies (1:5000, 711-035-152, Jackson Immunoresearch, West Grove, PA, USA) for 1 h. Then, the membranes were washed 3 times in TBST, and the HRP signal was detected by the ECL substrate (Bio-Rad) using the ImageQuant LAS 500 imaging system (GE Healthcare, Chicago, IL, USA). After analysis of phosphorylation of target proteins, membranes were stripped and re-probed for GAPDH with the rabbit anti-GAPDH antibody (1:1000, GB15004, ServiceBio) as described above. The results were analyzed using the ImageQuant TL 8.0 software (GE Healthcare) and the level of phosphorylated proteins was normalized to the GAPDH level. Absence of the HRP signal after membrane stripping was confirmed by imaging after 1 h of incubation with the secondary antibodies.

### 2.6. Flow Cytometry

To analyze GFAP expression in the primary astrocytes, the cells seeded on glasses in a 6-well plate (10 × 10^3^ cells per well) for 24 h were detached by the Versene solution (PanEco), fixed in 4% paraformaldehyde (PanEco) and incubated with the rabbit primary antibodies to GFAP (1:500, ABIN3043832, Antibodies-online, Aachen, Germany) for 1 h, then washed with PBS and incubated with anti-rabbit Alexa-488-conjugated antibodies (1:1000, 611-545-215, Jackson Immunoresearch).

To determine the effect of EVs on expression of cadherins on the glial cells’ membrane, cells (3 × 10^5^ cells per well of a 6-well plate) were treated with EVs (50 µg/mL) for 72 h, detached by the Versene solution, fixed in 4% paraformaldehyde, and incubated for 1 h with the rabbit primary antibodies to E cadherin (1:2000, 3195, Cell Signaling, Danvers, MA, USA), N cadherin (1:2000, 13116, Cell Signaling), or P cadherin (1:2000, 2189, Cell Signaling). The cells were then washed with PBS and incubated with the anti-rabbit Alexa-488-conjugated antibodies (1:1000, 611-545-215, Jackson Immunoresearch).

Cells stained only with the secondary antibodies were used as a negative control. All cells were analyzed by the Attune NxT flow cytometer (Life Technologies, Waltham, CA, USA) using the Attune NxT 2.3. Software (Life Technologies). The gating strategy is shown in Figure S2a.

### 2.7. Confocal Microscopy

Cells were seeded on glasses in a 24-well plate (3 × 10^3^ cells per well), incubated with EVs (50 µg/mL) for 72 h, blocked with 3% BSA for 16 h, stained with the rabbit antibodies to SNAI1 (1:200, FNab08051, FineTest, Wuhan, China) for 6 h, washed 3 times with PBS, and stained with the anti-rabbit Alexa-488-conjugated antibodies (1:1000, 611-545-215, Jackson Immunoresearch) for 1 h. After that, the glasses were blocked with 3% BSA for 16 h, stained with the rabbit antibodies to SNAI2 (1:200, FNab08052, FineTest) for 6 h, washed 3 times and stained with the anti-rabbit Alexa-647-conjugated antibodies (1:1000, 611-605-215, Jackson Immunoresearch) for 1 h. The cells were then washed 3 times, and nuclei were stained with Hoechst 33342. Glasses were embedded in the Mowiol-DABCO and examined under ×60 (1.4) objective of the Carl Zeiss LSM 710 confocal microscope (Carl Zeiss, Jena, Germany). The % of laser power and detector voltage were maintained constant. The number of SNAI clusters in the nuclei was analyzed using the Focinator 2.31 tool [56] in the ImageJ 1.54f software.

### 2.8. Analysis of Inflammation and Adhesion Regulators Secretion

To investigate the effect of EVs on the secretion of cytokines and adhesion factors by the astrocytes and GB cells, the 13-plex adhesion molecule panel immunoassay kit (740946, BioLegend, San Diego, CA, USA) was used. Cells were seeded on glasses in a 24-well plate (3 × 10^3^ cells per well), incubated with EVs (50 µg/mL) for 72 h, then 25 μL of cell media was collected from the untreated cells (control) or from the astrocytes incubated with EVs, GBM 011 and U251 MG cells and assayed in accordance with the manufacturer’s protocol. The results were analyzed by the Attune NxT flow cytometer (Life Technologies) using the Attune NxT Software 2.3. (Life Technologies). The calibration curve (5-parameter non-linear regression) built in the GraphPad 8.0 software (San Diego, CA, USA) was used to determine the concentration of substances. The calibration curves were obtained from [57]. The gating strategy is shown in Figure S2b.

### 2.9. Statistical Analysis

Data are presented as mean ± SEM. The number of samples (biological replicates, n) and statistical tests are indicated in the figure legends. Biological replicates were chosen by availability of the plasma for isolation of EVs. No exclusion criteria were applied to the experimental data, except for Western blotting. For Western blotting experiments, some bands were excluded due to technical failure. Before comparisons, the data were tested for normality (Shapiro–Wilk test, at *p* = 0.05). Differences in the data were considered statistically significant at *p* < 0.05. Analysis was performed using the GraphPad Prism 8.0 software.

## 3. Results

### 3.1. aEVs and bEVs from the Plasma of GB Patients Enhance Invasion of GB Cells

Since EVs from the cultured GB cells enhance invasion of GB cells [11], we studied whether EVs derived from GB patients crossing the BBB and circulating in the bloodstream influence invasion of GB cells. For this purpose, we used EVs from the plasma of GB patients isolated before and after tumor resection (bEVs and aEVs, respectively) and EVs isolated from the plasma of healthy donors (hEVs) as a control for the ‘general’ effect of plasma EVs. Analysis of the EV effects was performed on the normal rat primary cortical astrocytes and two GB cell lines: patient-derived glioblastoma GBM 011 cells and U251 MG cells.

**Figure 2:**
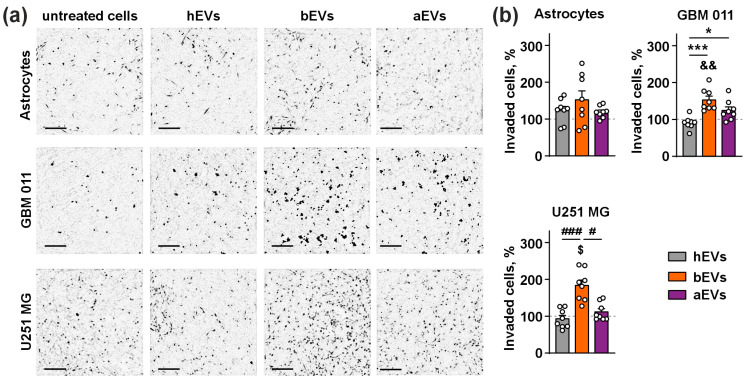
Influence of EVs on invasion of astrocytes, GBM 011, and U251 MG cells. (**a**) Representative images showing the cells migrated through 8 µM pore to the bottom of the migration chamber upon incubation with EVs (×100 magnification, scale bar = 100 μm); (**b**) number of the invaded cells. Data are shown as the number of invaded cells upon incubation with EVs normalized to the number of invaded untreated cells (100%, dashed line) ± SEM (n = 8); && (*p* < 0.01) indicates significant difference from the untreated cells according to one-sample *t*-test followed by post hoc Holm–Sidak’s test; $ (*p* < 0.05) indicates significant difference from the untreated cells according to one-sample Wilcoxon test, followed by post hoc Holm–Sidak’s test; * (*p* < 0.05) and *** (*p* < 0.001) indicate significant difference between the data groups according to one-way ANOVA followed by post hoc Tukey’s test; # (*p* < 0.05) and ### (*p* < 0.001) indicate significant difference between the data groups according to Kruskal–Wallis test followed by post hoc Dunn’s test.

The transwell assay showed that all types of EVs did not alter the motility of the normal astrocytes in comparison to the untreated cells, and no influence of hEVs on invasion of both astrocytes and GB cells was found (Figure 2). In the case of GBM 011 cells, bEVs increased cell invasion in comparison to the untreated cells, and bEVs and aEVs increased cell invasion by ~70% and 40% in comparison to hEVs, respectively (Figure 2). Also, bEVs increased the invasion of U251 MG cells in comparison to the cells treated with hEVs and untreated cells. Unlike GBM 011 cells, aEVs did not influence U251 MG cell invasion (Figure 2). We also compared the effect of aEVs, bEVs, and hEVs with EVs isolated from U251 MG cells on the invasion of U251 MG cells. We found that, similar to bEVs, EVs secreted by U251 MG cells increased the invasion of U251 MG cells (Figure S3). This points to the specific action of EVs on GB cell’s invasion.

### 3.2. aEVs and bEVs Activate MAP Kinases in GB Cells

**Figure 3:**
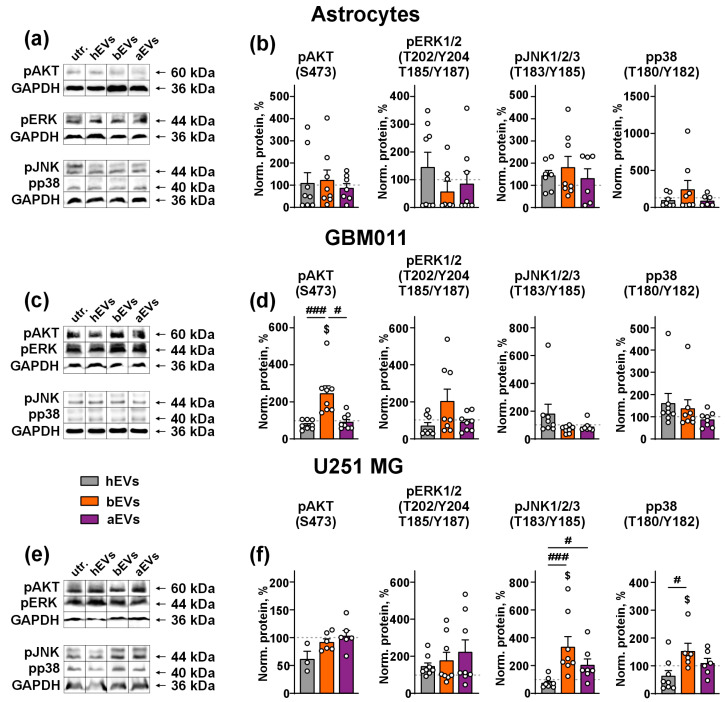
Analysis of influence of EVs on the MAP kinases phosphorylation in astrocytes (**a**,**b**), GBM 011 cells (**c**,**d**), and U251 MG cells (**e**,**f**). The following phosphorylation sites were assayed: pAKT (S473), pERK1/2 (T202/204; T185/Y187), pJNK1/2/3 (T183/Y185), and pp38 (T180/Y182). (**a**,**c**,**e**) Cut representative membranes with the bands (marked by frames); (**b**,**d**,**f**) quantification of the band intensity reflecting expression of different MAP kinases in the phosphorylated form. Please note that MAP kinases were assayed on the same (pJNK and pp38) or cut (pAKT and pERK) membranes, so they ‘share’ GAPDH. Whole Western blotting membranes are shown in Figures S4–S6. The quantification data are presented as band intensity (relative to GAPDH) normalized to that of the untreated cells (100%, dashed line) ± SEM (n = 3–8); $ (*p* < 0.05) indicates significant difference from the untreated cells according to one-sample Wilcoxon test, followed by post hoc Holm–Sidak’s test; # (*p* < 0.05) and ### (*p* < 0.001) indicate significant difference between the data groups according to Kruskal–Wallis test followed by post hoc Dunn’s test.

To investigate the molecular mechanisms underlying the effect of EVs on the invasion of GB cells, we examined the phosphorylation of key pro-migratory MAP kinases: AKT, ERK, JNK, and p38 by Western blotting. In the astrocytes, no type of EVs influenced the phosphorylation of MAP kinases (Figure 3a,b). In GBM 011 cells, bEVs up-regulated the phosphorylation of AKT (S437) by ~three-fold compared to hEVs. Furthermore, bEVs up-regulated the phosphorylation of AKT in relation to the untreated cells (Figure 3c,d). In U251 MG cells, bEVs significantly up-regulated the phosphorylation of JNK1/2/3 (T183/Y185) and p38 (T180/Y182) compared both to the untreated cells and cells treated with hEVs, while aEVs increased the phosphorylation only of JNK1/2/3 (T183/Y185) in comparison with hEVs (Figure 3e,f). hEVs did not activate any MAP kinases in any of the cell types studied.

### 3.3. aEVs and bEVs Drive Localization of SNAI1 to the Nuclei in GB Cells

**Figure 4:**
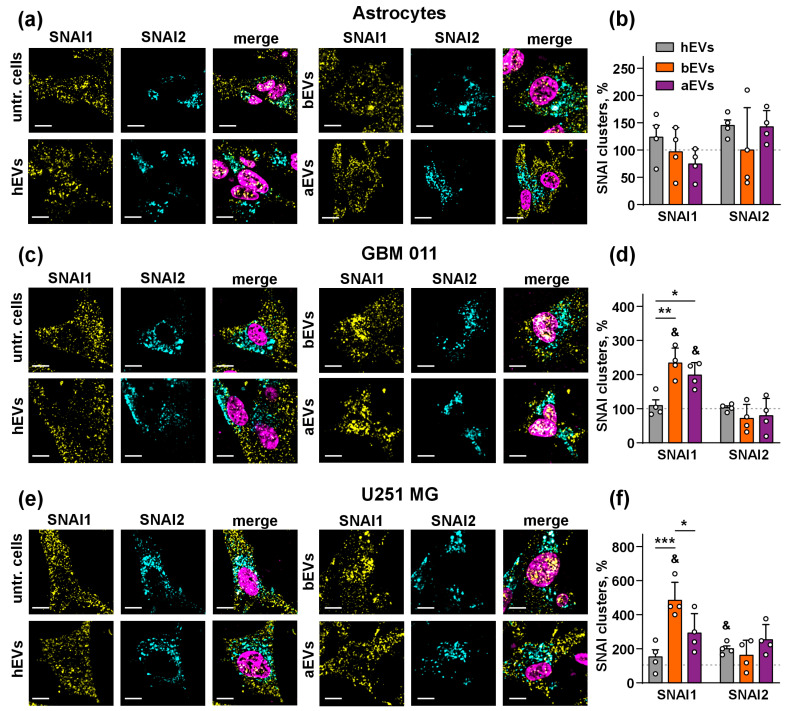
Influence of EVs on nuclear localization of SNAI1 and SNAI2 in astrocytes (**a**,**b**), GBM 011 cells (**c**,**d**), and U251 MG cells (**e**,**f**). Representative images of EV-treated astrocytes (**a**), GBM 011 cells (**c**), and U251 MG cells (**e**). Scale bar = 10 µm. (**b**,**d**,**f**) Number of SNAI clusters in the nuclei of studied cells. Data presented as a number of the clusters per the nuclei normalized to that of the untreated cells (100%, dashed line) ± SEM (n = 4 glasses, 3–4 cells from each glass were analyzed and averaged). & (*p* < 0.05) indicates significant difference from the untreated cells according to one-sample *t*-test followed by post hoc Holm–Sidak’s test; * (*p* < 0.05), ** (*p* < 0.01), and *** (*p* < 0.001) indicate significant difference between the data groups according to two-way ANOVA followed by post hoc Bonferroni’s test.

Previous studies proposed that the activation of AKT [58,59], JNK [59], and p38 [60] MAP kinases is associated with the activity of SNAI1 or SNAI2 zinc fingers, —the transcriptional factors that up-regulate cell migration and induce EMT in GB cells [61,62]. To study whether these factors are involved in the pro-invasive activity of aEVs and bEVs, we analyzed the localization of SNAI1 and SNAI2 in the nuclei of glial cells using confocal microscopy. hEVs were used as a control because they did not affect invasion of the astrocytes and GB cells and did not influence the activity of MAP kinases (Figure 2 and Figure 3). We found that all types of EVs did not affect the number of SNAI1 and SNAI2 clusters in the nuclei of the normal astrocytes (Figure 4a,b). However, we observed a significant increase in the number of the SNAI1 clusters in the nuclei of GBM 011 and U251 MG cells treated by bEVs (more than two times in both cases, Figure 4c–f). Treatment by aEVs led to an increase in the number of SNAI1 clusters in the nuclei of GBM 011 but not of U251 MG cells, although some non-significant increase was observed in the last case too (Figure 4c–f). A significant increase in the SNAI2 level in the nuclei was observed only for hEVs in U251 MG cells (Figure 4a–f).

### 3.4. aEVs and bEVs Induce E/N Cadherin Switch in GB Cells

Different cadherin isoforms regulate epithelial-mesenchymal transition (EMT) and invasion of GB cells [63]. Here, we investigated the influence of EVs on expression of different cadherins on the surface of the astrocytes and GB cells.

**Figure 5:**
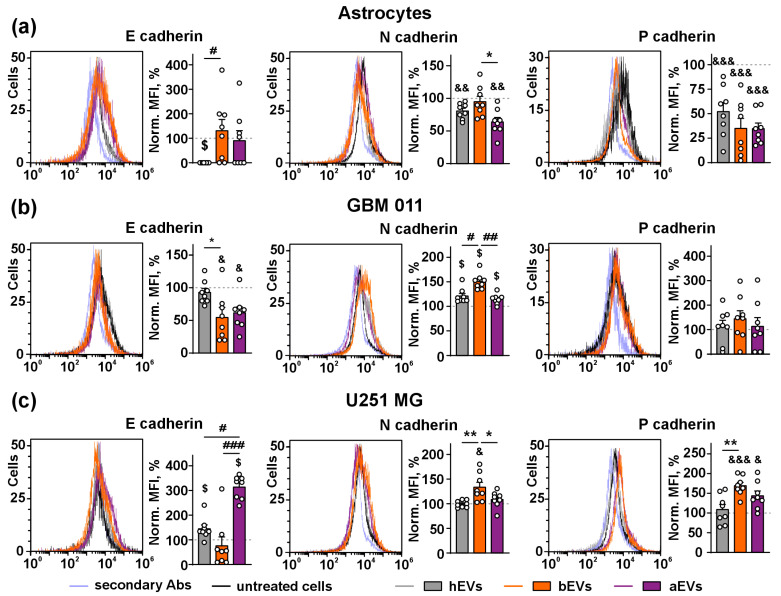
Influence of EVs on expression of E, N, and P cadherins on the surface of astrocytes (**a**), GBM 011 cells (**b**), and U251 MG cells (**c**). Representative cell distribution histograms and quantification of expression are shown on the left and right panels of the figures, respectively. The data are presented as background-subtracted MFI normalized to that of the untreated cells (100%, dashed line) ± SEM (n = 8). & (*p* < 0.05), && (*p* < 0.01), and &&& (*p* < 0.001) indicate significant difference from the untreated cells according to one-sample *t*-test followed by post hoc Holm–Sidak’s test; $ (*p* < 0.05) indicates significant difference from the untreated cells according to one-sample Wilcoxon test, followed by post hoc Holm–Sidak’s test; * (*p* < 0.05) and ** (*p* < 0.01) indicate significant difference between the data groups according to one-way ANOVA followed by post hoc Tukey’s test; # (*p* < 0.05), ## (*p* < 0.01), and ### (*p* < 0.001) indicate significant difference between the data groups according to Kruskal–Wallis test followed by post hoc Dunn’s test.

In the astrocytes, hEVs dramatically down-regulated expression of anti-migratory E cadherin to the zero level, while bEVs and aEVs did not affect its expression compared to the untreated cells (Figure 5a). The level of pro-migratory N cadherin in the astrocytes treated by hEVs and aEVs was lower by ~20–30% in relation to the untreated astrocytes, while bEVs did not demonstrate any effect (Figure 5a). All types of EVs significantly down-regulated by ~60–70% pro-invasive P cadherin expression in the astrocytes compared to the untreated cells, while no difference between the action of different EVs was found (Figure 5a).

In GBM 011 cells, both bEVs and aEVs down-regulated E cadherin expression in comparison to the untreated or hEV-treated cells (by ~50% in both cases, Figure 5b). At the same time, all types of EVs significantly increased expression of N cadherin in comparison to the untreated cells, and bEVs additionally up-regulated N cadherin expression by ~20% in comparison to the hEV-treated cells (Figure 5b). No effect of any types of EVs on P cadherin expression compared to the untreated cells was observed (Figure 5b).

In the U251 MG cells, hEVs and aEVs both up-regulated E cadherin expression compared to the untreated cells, and aEVs increased E cadherin expression 3-fold in comparison to hEV treated cells. No effect of bEVs on E cadherin expression was revealed (Figure 5c), while bEVs slightly but significantly up-regulated N-cadherin expression (by ~15%) in comparison to the hEV-treated, aEV-treated, and untreated cells. Similar to hEVs, aEVs did not affect N-cadherin expression (Figure 5c). Both bEVs and aEVs but not hEVs increased P cadherin expression in U251 MG cells (by ~40–50%, Figure 5c).

### 3.5. aEVs and bEVs Stimulate Secretion of Inflammation and Adhesion Regulators by GB Cells

EMT in GB is mediated by an establishment of immunosuppressive milieu, which is promoted by secretion of different cytokines and adhesion factors [13,14,15]. Using the multiplex immunoassay, we studied secretion of 13 cytokines and adhesion molecules by the astrocytes, GBM 011 cells, and U251 MG cells upon incubation with EVs and found that all types of them (hEVs, aEVs, and bEVs) affect secretion of many of the regulatory molecules by GB cells (Table S1).

**Figure 6:**
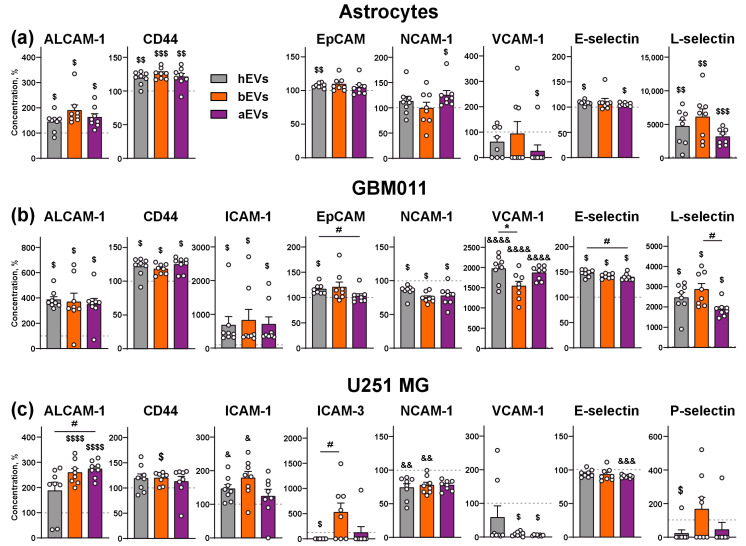
Influence of EVs on secretion of inflammation and adhesion regulators by astrocytes (**a**), GBM 011 cells (**b**), and U251 MG cells (**c**). The data are presented as molecules’ concentration in the cell growth media normalized to that of the untreated cells (100%, dashed line) ± SEM (n = 8). & (*p* < 0.05), && (*p* < 0.01), &&& (*p* < 0.001), and &&&& (*p* < 0.0001) indicate significant difference from the untreated cells according to one-sample *t*-test followed by post hoc Holm–Sidak’s test; $ (*p* < 0.05), $$ (*p* < 0.01), $$$ (*p* < 0.001), and $$$$ (*p* < 0.0001) indicate significant difference from the untreated cells according to one-sample Wilcoxon test, followed by post hoc Holm–Sidak’s test; * (*p* < 0.05) indicates significant difference between the data groups according to one-way ANOVA followed by post hoc Tukey’s test; # (*p* < 0.05) indicates significant difference between the data groups according to Kruskal–Wallis test followed by post hoc Dunn’s test.

In the astrocytes, all studied EVs up-regulated by ~50% secretion of pro-inflammatory factor ALCAM-1 (activated leukocyte cell adhesion molecule), and pro-migratory hyaluronic acid receptor CD44 (by ~25% Figure 6a, Table S1). hEVs slightly but significantly up-regulated secretion of EpCAM (epithelial cell adhesion molecule), which serves as a pro-inflammatory and pro-migratory cell junction protein. aEVs up-regulated by ~25% secretion of adhesion molecule NCAM-1 (neural cell adhesion molecule 1, and down-regulated by 3 times secretion of pro-migratory and anti-inflammatory factor VCAM-1 (vascular cell adhesion molecule 1). Both aEVs and hEVs slightly increased secretion of E-selectin (GB angiogenic modulator) by astrocytes (Figure 6a, Table S1). Untreated astrocytes did not secrete pro-inflammatory factor L-selectin, but all types of EVs caused secretion of this molecule (Figure 6a, Table S1).

In GBM011 cells, all types of studied EVs further up-regulated secretion of ALCAM-1 by 4-fold, CD44 (by ~20–25%), dramatically increased level of pro-inflammatory factor ICAM-1 (intercellular adhesion molecule 1), as well as E- and L-selectins (Figure 6b, Table S1). E-selectin was up-regulated by ~40–50% upon incubation with all types of EVs, while the effect of aEVs was lower than that of hEVs (Figure 6b). Secreted L-selectin was not detected in the media of the untreated cells, but all EVs dramatically up-regulated its secretion. Notably, the aEVs effect was significantly lower than that of bEVs (Figure 6b). Similar to the astrocytes, the level of secreted EpCAM in GBM011 cells was slightly increased upon incubation only with hEVs (Figure 6b). All types of EVs caused down-regulation of NCAM-1 secretion by ~15–25% (Figure 6b). Untreated GBM 011 cells secreted non-detectable amounts of VCAM-1, while all types of EVs caused dramatic up-regulation of its secretion, and hEVs demonstrated the stronger effect than bEVs (Figure 6b, Table S1).

In U251 MG cells, secretion of ALCAM-1 was significantly up-regulated upon incubation with aEVs and bEVs in comparison with the untreated cells. hEVs also stimulated secretion of this factor, although the difference with the untreated cells did not reach statistical significance. Notably, effect of aEVs was significantly higher in comparison to hEVs (Figure 6c). bEVs up-regulated by 20 CD44 secretion in comparison to the untreated cells (Figure 6c, Table S1). hEVs and bEVs increased secretion of ICAM-1 (by ~150–200%) and down-regulated the level of NCAM-1 (by ~25%) in comparison to untreated cells. aEVs also diminished secretion of NCAM-1, but the difference with the untread cells did not reach the significance (Figure 6c, Table S1). bEVs increased by 5-fold secretion of pro-inflammatory apoptosis inhibitor ICAM-3 (intercellular adhesion molecule 3), while hEVs down-regulated secretion of this molecule up to the zero level and aEVs did not affect its secretion (Figure 6c, Table S1). Moreover, aEVs and bEVs dramatically decreased secretion of VCAM-1 (Figure 6c, Table S1). Unlike to bEVs and hEVs, aEVs slightly decreased the E-selectin secreted level, while hEVs down-regulated secretion of adhesion regulator P-selectin (Figure 6c, Table S1).

## 4. Discussion

GDEVs are the main molecule carriers between tumors and their microenvironment [5]. Despite an ability of EVs to cross the BBB and circulate in the bloodstream [6], little is known about the influence of GB vesicles from the bloodstream on homeostasis of the normal astrocytes and GB cells. To fill this gap, we performed a comparative analysis of the effects of EVs from the plasma of healthy donors (hEVs) and GB patients (bEVs and aEVs) in the normal astrocytes and GB cells.

First, we analyzed the influence of the vesicles on cell invasion and found that hEVs did not affect the invasion of either astrocytes or GB cells. At the same time, bEVs increased the invasion both of GBM 011 and U251 MG cells, and aEVs increased the invasion of GBM 011 cells (Figure 2). It has previously been shown that EVs secreted by model GB cells stimulate invasion of GB cells [4,64], but here we showed for the first time that EVs isolated from the bloodstream of GB patients also stimulate invasion of the GB cells. The fact that aEVs induce GB cell invasion shows that even after tumor resection, residual GB cells can be re-stimulated to spread along the body by aEVs remaining in the bloodstream. Thus, GDEVs may mediate the formation of secondary cancers in the GB patients, which are known to occur in long-term GB survivors [65].

We showed that bEVs activate in the GB cells several MAP kinases: AKT and ERK in GBM 011 cells and JNK with p38 in U251 MG cells, while aEVs increase the phosphorylation level only of JNK in U251 MG cells in comparison with hEVs (Figure 3). JNK activation promotes GB stem cell invasion [66], while p38 phosphorylation promotes invasion of glioma cells [67]. Thus, their activation in U251 MG cells is consistent with the pro-invasive activity of aEVs and bEVs. Notably, no influence on the activity of MAP kinases was observed for all EVs studied here in the normal astrocytes (Figure 3). Interestingly, in spite of absence of the broad effect on the kinase activity, aEVs still retain their pro-invasive activity in GBM 011 cells. This means that aEVs and bEVs may mediate their pro-invasive effect through different molecular mechanisms.

Here we detected the increased number of the SNAI1 but not of SNAI2 clusters in the nuclei of the GB cells upon incubation with aEVs and bEVs, and this effect was not observed in the normal astrocytes (Figure 4). It should be noted that SNAI1 is the transcriptional repressor [68], and its localization to the cell nucleus is required to inhibit expression of the anti-migratory factors, such as the cell junction protein E cadherin [69]. In line with this, we showed that aEVs and bEVs induce the E/N cadherin switch: down-regulation of surface expression of E cadherin with simultaneous up-regulation of N cadherin expression in the GB cells (Figure 5). The E/N cadherin switch is the common marker of EMT [70], which is required for GB metastasis and invasion [71]. EMT can be influenced by the GB–astrocyte communication [71], and our data propose that aEVs and bEVs may modulate the GB–astrocyte interaction via the E/N cadherin switch. Induction of the E/N cadherin switch by aEVs in GBM 011 cells (although not so strongly as by bEVs, Figure 5b) additionally points to possible remaining of invasion stimulating effects even after tumor resection.

Interestingly, hEVs down-regulated both N and E cadherins in the astrocytes, suggesting that hEVs may also regulate the formation of intercellular contacts in the normal astrocytes under heathy conditions. It should be noted that up-regulation of E cadherin observed here for aEVs in U251 MG cells (Figure 5c) was previously described in GB patients and xenografts and is associated with tumor invasion [72]. P cadherin drives GB proliferation, migration, and invasion and is considered a biomarker of GB [73]. Its up-regulation in U251 MG cells (Figure 5c) may mediate the pro-invasive effect of bEVS.

GB progression is accompanied by the secretion of many inflammatory and pro-migratory factors in the tumor microenvironment [2]. EVs can stimulate secretion of some cytokines and growth factors by the GB cells and astrocytes [2,15]. Here, we found that all types of EVs influence secretion of many adhesion molecules, some of which regulate GB cell migration (Figure 6, Table S1). In the normal astrocytes, GMB011, and U251 MG cells, all types of EVs activated the secretion of ALCAM-1, the pro-inflammatory factor [74], which stimulates GB invasion in vivo and whose level correlates with a worse survival prognosis for GB patients [75]. CD44, which was up-regulated in the astrocytes and GB cells upon incubation with EVs, induces GB cell growth and invasiveness [76,77]. Secretion of the pro-inflammatory cytokine EpCAM [78], which was up-regulated in the astrocytes and GBM011 cells upon incubation with hEVs, drives carcinoma invasion but its role in GB progression is not well understood [79]. ICAM-1, which was absent in the media of the astrocytes but was up-regulated in the media of the GB cells upon treatment with all types of EVs (Figure 6, Table S1), is a pro-inflammatory factor that stimulates invasiveness of chemotherapy-resistant GB cells in vivo [80]. ICAM-3, whose secretion was up-regulated only by bEVs in U251 MG cells, drives carcinoma cell migration and invasion via the AKT pathway [81,82]. Notably, bEVs stimulated the AKT activity in GB011 cells; although for U251 MG cells, this effect was not found (Figure 3). Nevertheless, we hypothesize that the pro-invasive activity of bEVs in U251 MG cells (which was not observed for aEVs) may be mediated by the ICAM-3-AKT functional interaction. NCAM-1 regulates the extracellular matrix degradation and serves as the inhibitor of GB cell invasion [83]. Its overexpression decreases GB cell motility [84]. Here, we observed either no effects from hEVs and bEVs and stimulation of NCAM-1 secretion by aEVs in the astrocytes or down-regulation of NCAM-1 in the GB cells upon incubation with all types of EVs (Figure 6, Table S1). This agrees well with the data on the stimulation of GB cell invasion by aEVs and bEVs (Figure 2).

VCAM-1 promotes tumor invasion and metastasis through interaction with tumor-associated macrophages [85]. Interestingly, VCAM-1 is also an immunosuppressive and anti-inflammatory factor, in particular, it reduces T-cell infiltration to carcinoma lesions [86]. Thus, it may not only promote GB cell invasion but also may mediate the formation of an immunosuppressive GB environment. Here, EVs differentially regulated VCAM-1 secretion: aEVs and bEVs decreased it in U251 MG cells (while hEVs demonstrated no effect), but in GBM011 cells, all EVs (including hEVs) dramatically increased VCAM-1 secretion. At the same time, only aEVs down-regulated its secretion in the astrocytes (Figure 6, Table S1). Pro-inflammatory E-, L-, and P-selectins [87] regulate leucocyte adhesion, so their up-regulation can be associated with formation of the tumor inflammatory microenvironment [88]. Selectins can mediate tumor cell invasion by facilitating the interaction of tumor cells with the endothelium [88], thus, they may also be involved in GB invasion. In addition, P-selectin induces secretion of anti-inflammatory cytokines by microglia, thus inhibiting anti-tumor immunity and promoting GB cell invasion [89]. In our work, a slight increase in E-selectin secretion was revealed in the astrocytes treated by aEVs and hEVs, while in GB011 cells, we observed its up-regulation by all types of EVs with a simultaneous close-to-zero effect from EVs in U251 MG cells. For L-selectin, dramatic up-regulation by all EVs was detected in both astrocytes and GB011 cells, while for P-selectin, insignificant up-regulation of its secretion was found only upon incubation of U251 MG cells with bEVs (Figure 6, Table S1). Such diverse effects of different EVs on secretion of various regulatory molecules may be associated with different factors (see below) and requires further study. However, in almost all the cases, a tendency to maintain a pro-inflammatory environment is observed.

Similar to plasma GDEVs, GDEVs from CSF can carry mRNA of pro-oncogenic factors such as the mutant variant of the EGFR, -*EGFRvIII* [20], and pro-oncogenic miRNAs [28,37,90]. Although, the effects of GDEVs from CSF on the GB cells were not previously studied, we propose that the effects of GDEVs from CSF on the invasion and regulation of protein expression/secretion by the normal and tumor cells can be similar to those observed here for plasma GDEVs. In line with this, both CSF and plasma GDEVs contain pro-oncogenic miR-21, miR-24, miR-103, and miR-125 [90]. Further study of GDEVs from CSF is necessary.

Different overall effects of GDEVs on the astrocytes, GBM 011 cells, and U251 MG cells should also be emphasized. In GBM011 but not in U251 MG cells, aEVs increased cell invasion. In addition, different MAP kinases were activated by bEVs in these cells: AKT in GBM011 cells and JNK and p38 in U251 MG cells. EVs also differentially regulate the secretion of cytokines and adhesion factors by the GB cells. We propose that the difference between these effects can be explained by several factors such as (i) different molecular characteristics of the GB cells, (ii) different localization of the tumors, (iii) the fact that we studied the effect of all plasma EVs but not of a specific EV population, and (iv) different genetics of tumor cells. High heterogeneity of GB tumors is well known [1]; thus, the effect of EVs may also be mediated by the characteristics of the GB cells.

It should be emphasized that hEVs also affected the normal astrocytes and GB cells: up-regulated SNAI2 level in the nuclei of U251 MG cells (Figure 4), down-regulated cadherins expression in the astrocytes, and up-regulated E and N cadherins expression in U251 MG and GBM011 cells, respectively (Figure 5). hEVs also up-regulated the secretion of cytokines and pro-invasion adhesion factors by the astrocytes and GB cells (Figure 6, Table S1). It was described that hEVs can transfer some pro-migratory factors and be implicated in the regulation of cell growth, migration, and angiogenesis [91]. Additionally, our study points to hEVs as the regulators of intercellular contacts and possible stimulators of pro-oncogenic responses in GB cells. The possible pro-oncogenic activity of hEVs should be further studied to ensure their safety for targeted delivery of therapeutic substances to GB.

**Figure 7:**
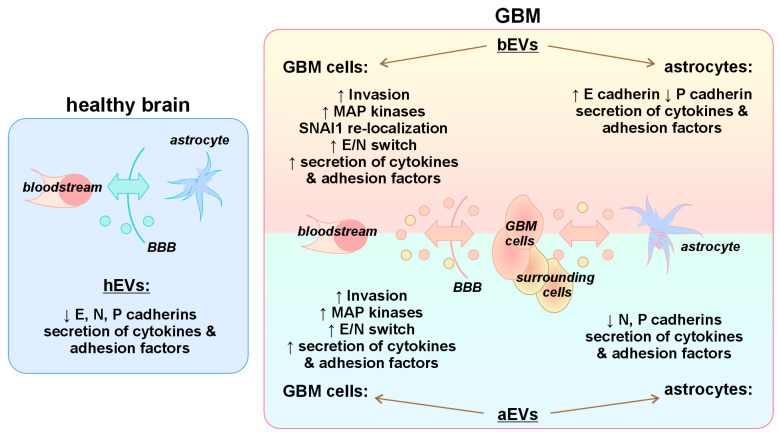
Proposed model of influence of EVs from the plasma of healthy donors and GB patients before and after tumor resection in astrocytes and GB cells. The effects revealed in our work for aEVs, bEVs, and hEVs are listed. ↑ and ↓ indicate up- and down-regulation of protein expression or secretion in different cells upon incubation with EVs.

In general, we can propose the model of EV activity in the astrocytes and GB cells (Figure 7). In health, hEVs crossing the BBB have very little effect on the normal astrocytes: they do not alter cell motility and MAP kinases’ activity. However, they down-regulate the expression of N and E cadherins, thus regulating the intercellular contacts. In GB, before tumor resection, bEVs are secreted by the tumor and surrounding cells providing the tumor progression. As vesicles can cross the BBB in both directions, some bEVs may return back to the tumor and increase GB invasion. This may be mediated by the activation of some invasion-related MAP kinases (such as AKT, ERK, JNK, or p38) and supplemented by increasing the amount of SNAI1 clusters in the tumor cell nucleus. In the nucleus, SNAI1 can repress the E cadherin transcription and promote the E/N cadherin switch,—the marker of EMT. In addition, bEVs increase the expression of pro-invasive P cadherin. Besides the induction of invasion, bEVs up-regulate the secretion of pro-migratory cytokines and adhesion factors. Interestingly, bEVs have no effect on the normal astrocytes. This is difficult to explain, but we can speculate that EVs only activate the astrocytes, making them reactive without malignant transformation. Another possibility is that the time of our in vitro experiments (72 h, chosen so that the cells did not die at the end of the experiment) is not enough for the astrocytic transformation. Moreover, we cannot exclude the existence of possible protective mechanisms in the astrocytes preventing their transformation.

When GB is resected, EVs previously secreted by the tumor and tumor surrounding cells remain in the bloodstream (aEVs), although their activity can be changed already on the next day after resection. As they can cross the BBB, they can return to the brain and affect the GB cells remaining after tumor resection. aEVs may mediate the invasion of GB cells by the activation of JNK and E/N switch, as well as by the stimulation of the secretion of different pro-migratory cytokines and inflammatory adhesion molecules in the remaining GB cells (Figure 7). Thus, even after tumor resection, aEVs can stimulate intercellular contacts of tumor cells and provide conditions for the tumor progression.

Nevertheless, our work has some limitations. In particular, we used rat astrocytes as a model of the normal astrocytes. It is not clear how many EVs return to the tumor after being secreted into the bloodstream and how fast tumorigenic activity of aEVs changes and in what direction after tumor resection. Some common effects of hEVs and GDEVs may overlap when we choose hEVs as a baseline for the analysis of the GDEVs’ effects. Moreover, we analyzed the effect of all EVs isolated from the plasma, including ones secreted by non-GB cells surrounding the tumor. Thus, some effects can be mediated not only by EVs from the GB tissue but also by tumor-surrounding cells. Also, an analysis of EVs isolated after a longer time period after the tumor resection can give us more information about the EV impact in GB recurrence. All mentioned points require additional investigation. Analysis of the content of EVs and study of the mechanisms of their action in the tumor and normal cells would improve our understanding of the EV role in the GB progression and recurrence. Another interesting issue raised from our results is the possible influence of plasma EVs on non-neuronal cells, which may help us to better understand the formation of secondary cancers in GB survivors.

## 5. Conclusions

Obtained results point to EVs as one of the most important factors in the GB progression and provide possible mechanisms by which EVs may not only promote GB invasion but also cause tumor recurrence. Overall, our study provides a landscape of plasma EV influence in health and during GB progression and shows that even after tumor resection, aEVs from the bloodstream can stimulate the remaining GB cells. This may either lead to GB recurrence or somehow affect other tissues in the body, causing secondary tumors in GB survivors. It is probable that clearing of the patient’s bloodstream from EVs after tumor resection may be a new prospective strategy to prevent GB recurrence or secondary cancers. Moreover, stimulation of some pro-oncogenic effects by hEVs in the GB cells, that requires additional attention when using hEVs in therapy and diagnostics, is described.

## Data Availability

All datasets generated within the experiment are available upon request.

## Supplementary Materials

The following supporting information can be downloaded at: https://www.mdpi.com/article/10.3390/biomedicines12122834/s1. Table S1. Concentrations of adhesion molecules assayed by the 13× adhesion panel LegendPlex immunoassay kit; Figure S1. Comparison of EVs isolated by ultracentrifugation and by the Total Exosome Isolation kit (from plasma); Figure S2. Representative gating strategy flow cytometry analyses; Figure S3. Comparison of action of EVs from U251 MG cells and GDEVs on invasion of U251 MG cells; Figure S4. Whole Western blotting membranes of pAKT, pERK (a), pJNK, and pp38 (b) expression in astrocytes; Figure S5. Whole Western blotting membranes of pAKT, pERK (a), pJNK, and pp38 (b) ex-pression in GBM 011 cells; Figure S6. Whole Western blotting membranes of pAKT, pERK (a), pJNK, and pp38 (b) expression in U251 MG cells.
