# Supplementary material for: Extracellular Vesicles from Plasma of Patients with Glioblastoma Promote Invasion of Glioblastoma Cells Even After Tumor Resection
Source: Biomedicines. 2024 Dec 13;12(12):2834. doi: 10.3390/biomedicines12122834 (PMC11673896; doi:10.3390/biomedicines12122834)
Supplement: Supplementary file 1 [file biomedicines-12-02834-s001.zip › Supplementary material.pdf]

biomedicines

MDPI

Supplementary information

Extracellular Vesicles from Plasma of Patients with Glioblastoma Promote Invasion of Glioblastoma Cells Even After Tumor Resection

Ekaterina N. Lyukmanova [1,2,3,4,*], Artem V. Kirichenko [2,3], Igor A. Medyanik [5], Konstantin S. Yashin [5], Mikhail P. Kirpichnikov [2,4] and Maxim L. Bychkov [2,*]

[1] Faculty of Biology, Shenzhen MSU-BIT University, Shenzhen 518172, China
[2] Shemyakin-Ovchinnikov Institute of Bioorganic Chemistry, Russian Academy of Sciences, 119997 Moscow, Russia; bittert@mail.ru (A.V.K.); kirpichnikov@inbox.ru (M.P.K.)
[3] Moscow Center for Advanced Studies, 123592 Moscow, Russia
[4] Interdisciplinary Scientific and Educational School of Moscow University «Molecular Technologies of the Living Systems and Synthetic Biology», Faculty of Biology, Lomonosov Moscow State University, 119234 Moscow, Russia
[5] Department of Neurosurgery, Privolzhsky Research Medical University, 603005 Nizhny Novgorod, Russia; med_neuro@inbox.ru (I.A.M.); jashinmed@gmail.com (K.S.Y.)
* Correspondence: lyukmanova_ekaterina@smbu.edu.cn (E.N.L.); maksim.bychkov@gmail.com (M.L.B.)

Table S1. Concentration of adhesion molecules assayed by the 13x adhesion panel LegendPlex immunoassay kit[a].

Cytokine	Concentration, ng/mL ± S.E.M.			
	Untreated cells	Treated by EVs		
		hEVs	bEVs	aEVs
		**Astrocytes**		
**ALCAM-1**	3,94 ± 0,33	5,67 ± 0,51$	7,47 ± 0,86$	6,39 ± 0,58$
**CD44**	66,26 ± 1,87	78,81 ± 2,31$$	83,31 ± 1,90$$$	80,14 ± 3,39$$
**EpCAM**	0,24 ± 0,004	0,26 ± 0,003$$	0,27 ± 0,009	0,25 ± 0,01
**ICAM-1**	0,06 ± 0,005	0,029 ± 0,01	0,12 ± 0,04	0,05 ± 0,01
**ICAM-2**	n.d.[b]	n.d.	n.d.	n.d.
**ICAM-3**	n.d.	n.d.	n.d.	n.d.
**NCAM-1**	59,42 ± 1,44	67,01 ± 5,92	58,88 ± 7,15	74,24 ± 5,57$
**VCAM-1**	0,11 ± 0,04	0,07 ± 0,02	0,07 ± 0,05	0,03 ± 0,03$
**PECAM-1**	0,14 ± 0,08	0,31 ± 0,07	0,25 ± 0,07	0,23 ± 0,09
**PSGL-1**	0,33 ± 0,01	0,33 ± 0,01	0,34 ± 0,01	0,33 ± 0,01
**E-selectin**	0,13 ± 0,002	0,14 ± 0,002$	0,14 ± 0,008	0,13 ± 0,001$
**L-selectin**	0.39 ± 0.3	18,51 ± 3,70$$	23,97 ± 4,79$$	12,43 ± 1,65$$$
**P-selectin**	n.d.	n.d.	n.d.	n.d.
		**GBM011 cells**		
**ALCAM-1**	3,31 ± 0,28	12,74 ± 0,83$	12,18 ± 2,31$	11,48 ± 1,65$
**CD44**	64,63 ± 0,67	78,45 ± 3,19$	76,24 ± 1,74$	80,47 ± 2,50$
**EpCAM**	0,25 ± 0,003	0,29 ± 0,008$	0,30 ± 0,03	0,25 ± 0,01
**ICAM-1**	0,05 ± 0,01	0,36 ± 0,14$	0,44 ± 0,17$	0,37 ± 0,12$
**ICAM-2**	n.d.	n.d.	n.d.	n.d.
**ICAM-3**	n.d.	n.d.	n.d.	n.d.
**NCAM-1**	58,86 ± 2,11	50,05 ± 1,76$	44,02 ± 1,56$	45,49 ± 3,05$
**VCAM-1**	0.78 ±0.78	14,34 ± 1,73&&&&	11,97 ± 0,92&&&&	14,50 ± 0,49&&&&
**PECAM-1**	0,02 ± 0.02	0,35 ± 0,10	0,45 ± 0,21	0,23 ± 0,09
**PSGL-1**	0,32 ± 0,005	0,30 ± 0,04	0,30 ± 0,009	0,30 ± 0,008
**E-selectin**	0,13 ± 0,003	0,19 ± 0,003$	0,18 ± 0,002$	0,18 ± 0,003$
**L-selectin**	0.99 ± 0.19	24,53 ± 2,60$	28,64 ± 2,90$	18,82 ± 1,23$
**P-selectin**	n.d.	n.d.	n.d.	n.d.
		**U251 MG cells**		
**ALCAM-1**	10,89 ± 4,19	20,33 ± 3,76	28,18 ± 1,83$$$$	29,73 ± 1,29$$$$
**CD44**	72,98 ± 4,54	86,62 ± 6,73	87,30 ± 3,09$	82,21 ± 7,67
**EpCAM**	0,27 ± 0.01	0,30 ± 0.01	0,31± 0.01	0,31 ± 0.007
**ICAM-1**	0,06 ± 0,02	0,09 ± 0,008&	0,12 ± 0,01&	0,08 ± 0,01
**ICAM-2**	n.d.	n.d.	n.d.	n.d.
**ICAM-3**	0.6 ± 0.4	n.d.$	3,14 ± 1,13	0,73 ± 0,73
**NCAM-1**	33,14 ± 5,73	24,48 ± 1,98&&	25,65 ± 1,51&&	25,65 ± 0,89
**VCAM-1**	5,87 ± 2,06	3,39 ± 2,05	0,52 ± 0,13$	0,30 ± 0,04$
**PECAM-1**	0,20 ± 0,08	0,22 ± 0,09	0,22 ± 0,12	0,059 ± 0,059
**PSGL-1**	0,33 ± 0,01	0,33 ± 0,02	0,35 ± 0,02	0,35 ± 0,01
**E-selectin**	0,17 ± 0,01	0,16 ± 0,003	0,16 ± 0,005	0,15 ± 0,001&&&
**L-selectin**	12,65 ± 4,79	15,19 ± 2,91	18,79 ± 2,10	20,94 ± 3,61
**P-selectin**	0,05 ± 0,03	0,01 ± 0,01$	0,09 ± 0,04	0,02 ± 0.02

[a]Please note, that in the main body of the article we discuss molecules, whose levels were changed between the groups treated by different EVs and in comparison with untreated cells.

[b]n.d. – not detected

& ($p < 0.05$), && ($p < 0.01$), &&& ($p < 0.001$), and &&&& ($p < 0.0001$) indicate significant difference from the untreated cells according to one-sample t-test followed by *post hoc* Holm-Sidak's test. $ ($p < 0.05$), $$ ($p < 0.01$), $$$ ($p < 0.001$), and $$$$ ($p < 0.0001$) indicate significant difference from the untreated cells according to one-sample Wilcoxon test, followed by *post hoc* Holm-Sidak's test.

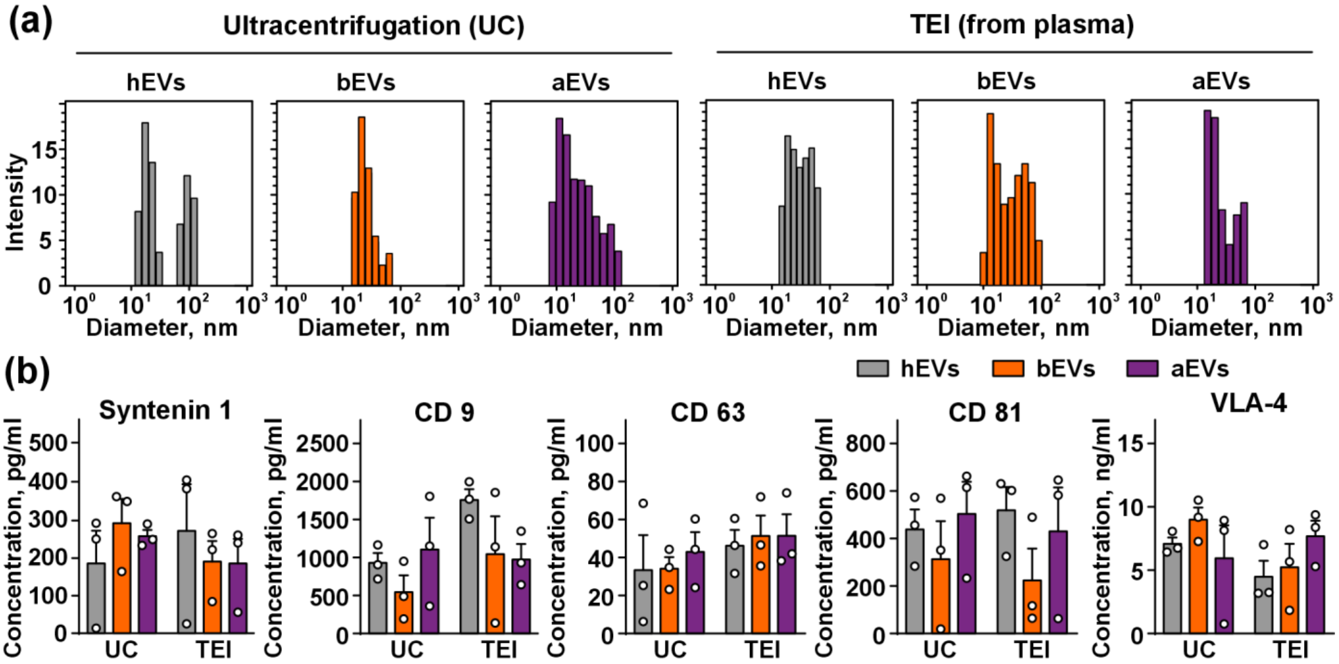

Figure S1. Comparison of EVs isolated by ultracentrifugation and by the Total Exosome Isolation Kit (from plasma). (a) Representative distribution of EVs according to the size determined by dynamic light scattering, (b) Expression of EVs markers (analyzed by ProcartaPlex™ Human Exosome Characterization Panel), no cytochrome C was detected in all studied EVs. The data are EVs marker concentration ± S.E.M. (n = 3). No difference between the data groups was found according to one-way ANOVA followed by *post hoc* Tukey's test.

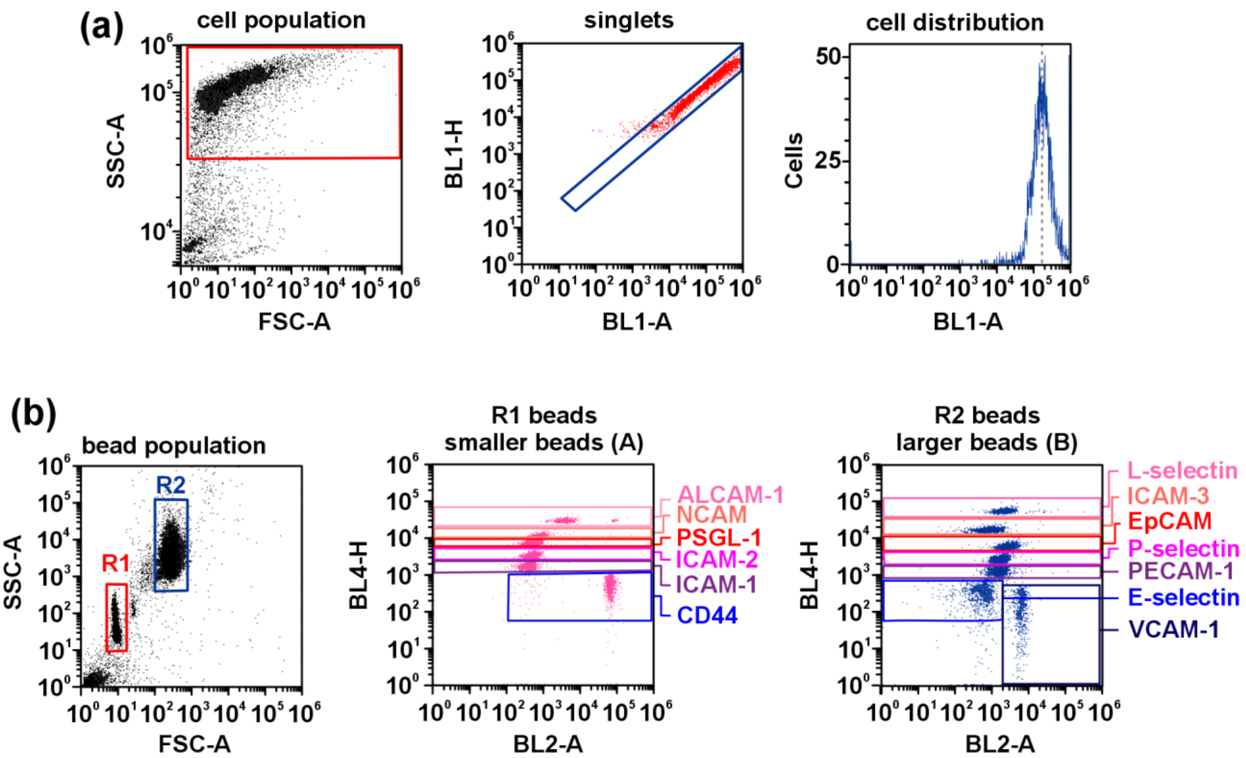

Figure S2. Representative gating strategy flow cytometry analyses: (a) for analysis of cadherins expression on the cell surface, (b) for analysis of adhesion molecules secretion by the astrocytes and GB cells (assayed by the 13x adhesion panel LegendPlex immunoassay kit).

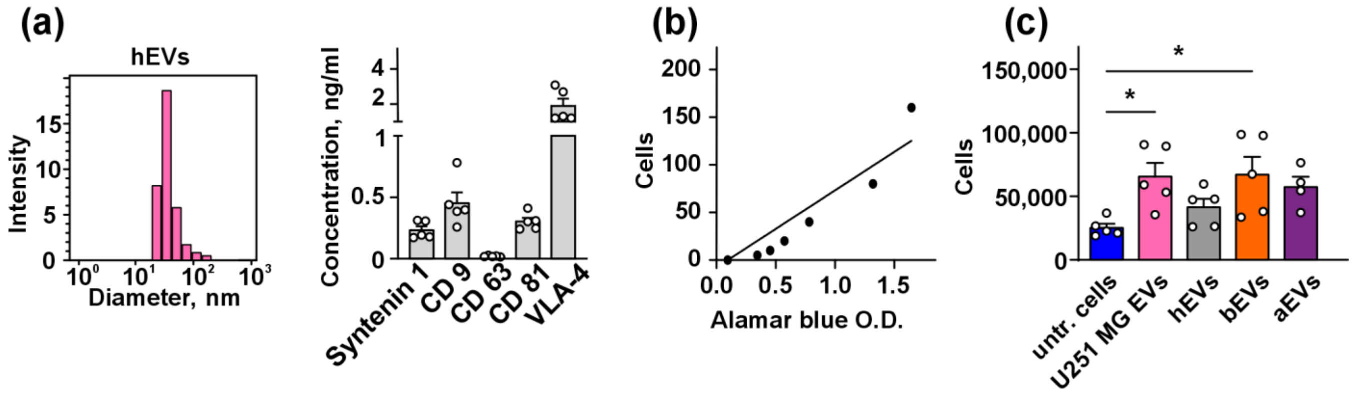

Figure S3. Comparison of action of EVs from U251 MG cells and GB EVs on invasion of U251 MG cells. (a) Characterization of EVs from U251MG cell' media by DLS (right) and ProcartaPlex™ Human Exosome Characterization Panel (left), no cytochrome c was detected. (b) Calibration curve used for Alamar blue assay. (c) Analysis of the number of invaded cells. Data presented as number of invaded cells, quantified from the calibration curve ± SEM (n = 5). * ($p < 0.05$) indicates significant difference between the data groups according to one-way ANOVA followed by *post hoc* Tukey's test.

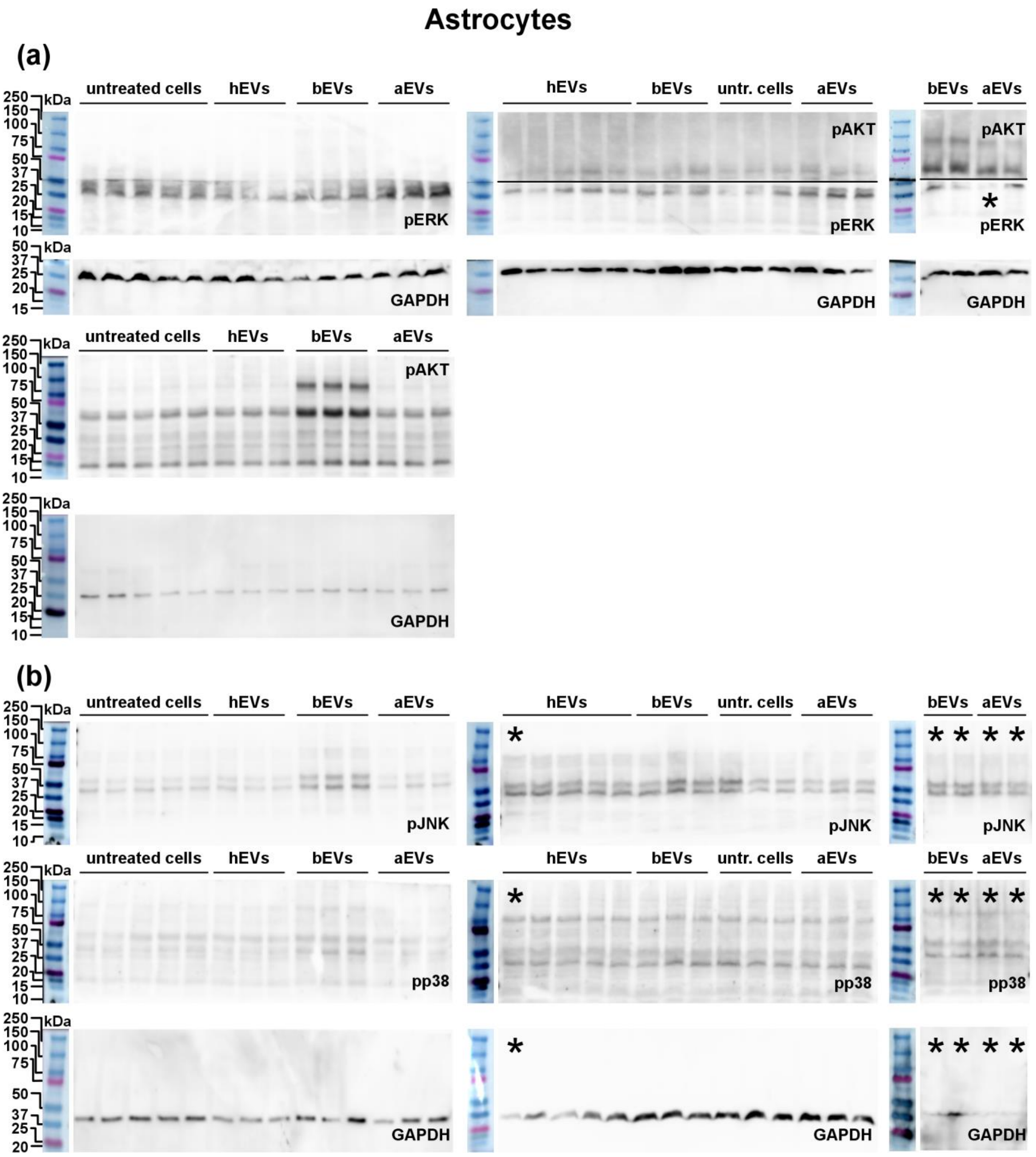

Figure S4. Whole Western blotting membranes of pAKT, pERK (a), pJNK, and pp38 (b) expression in the astrocytes. The protein level (band intensity) was normalized to the level of GAPDH. * indicates probe excluded due to technical fail.

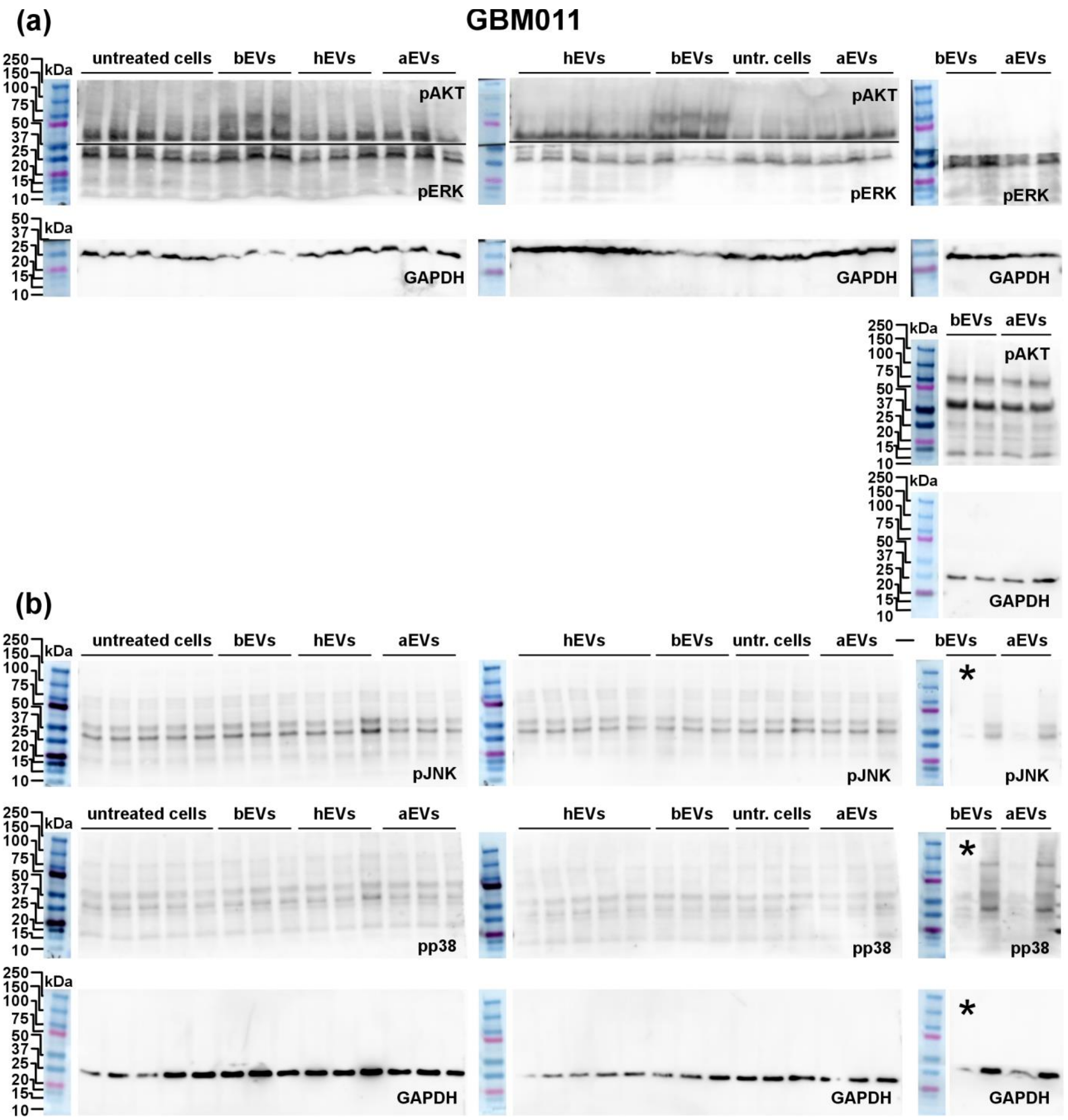

Figure S5. Whole Western blotting membranes of pAKT, pERK (a), pJNK, and pp38 (b) expression in the GBM 011 cells. The protein level (band intensity) was normalized to the level of GAPDH. * indicates probe excluded due to technical fail.

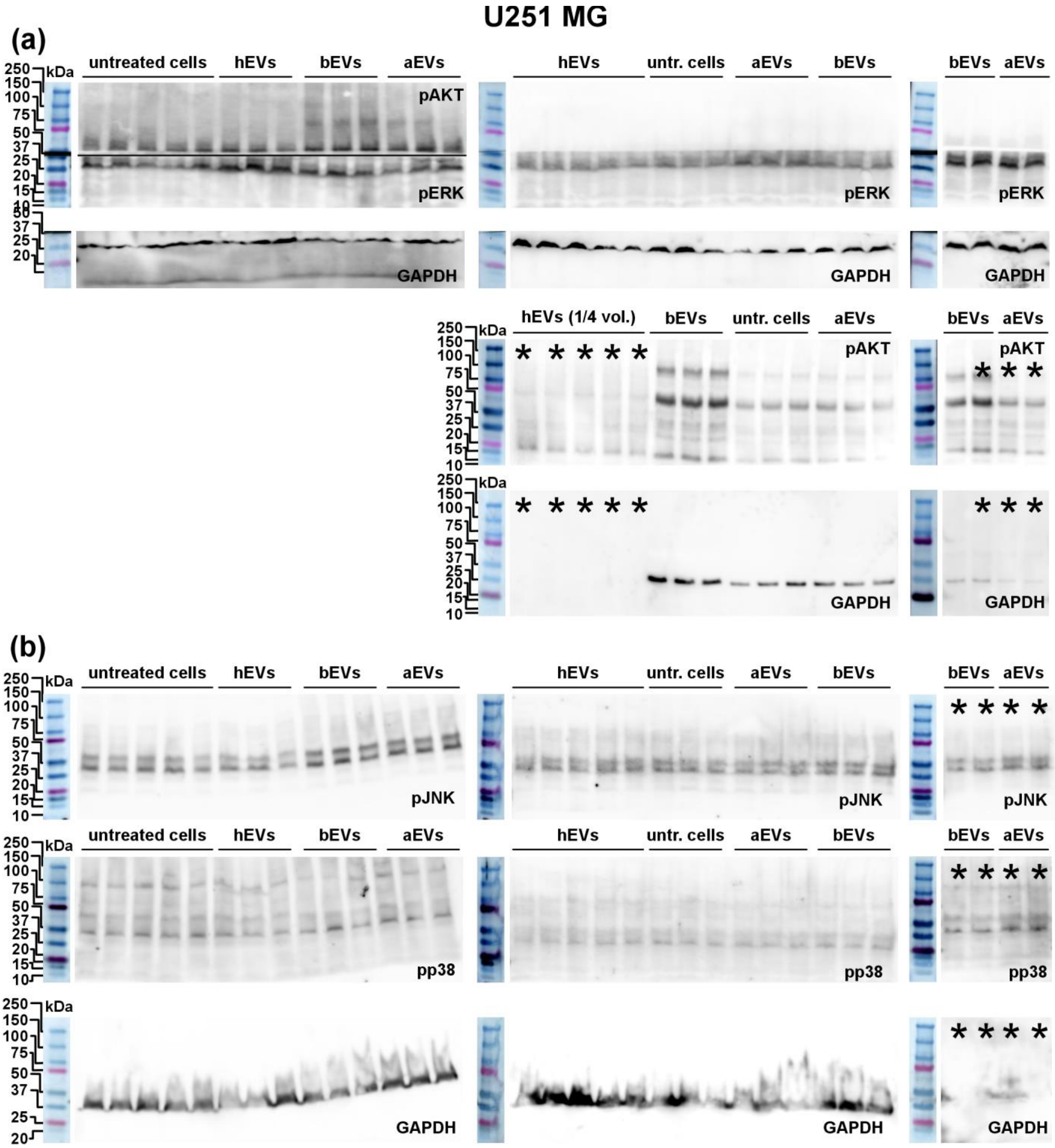

Figure S6. Whole Western blotting membranes of pAKT, pERK (a), pJNK, and pp38 (b) expression in the U251 MG cells. The protein level (band intensity) was normalized to the level of GAPDH. * indicates probe excluded due to technical fail.
